# Determination of the human spine curve based on laser triangulation

**DOI:** 10.1186/s12880-015-0044-5

**Published:** 2015-02-05

**Authors:** Primož Poredoš, Dušan Čelan, Janez Možina, Matija Jezeršek

**Affiliations:** University of Ljubljana, Faculty of Mechanical Engineering, Aškerčeva 6, 1000 Ljubljana, Slovenia; University Medical Centre Maribor, Ljubljanska ulica 5, 2000 Maribor, Slovenia

**Keywords:** Laser profilometry, 3D, Back shape analysis, Scoliosis, Spatial spine curve, Cubic splines

## Abstract

**Background:**

The main objective of the present method was to automatically obtain a spatial curve of the thoracic and lumbar spine based on a 3D shape measurement of a human torso with developed scoliosis. Manual determination of the spine curve, which was based on palpation of the thoracic and lumbar spinous processes, was found to be an appropriate way to validate the method. Therefore a new, noninvasive, optical 3D method for human torso evaluation in medical practice is introduced.

**Methods:**

Twenty-four patients with confirmed clinical diagnosis of scoliosis were scanned using a specially developed 3D laser profilometer. The measuring principle of the system is based on laser triangulation with one-laser-plane illumination. The measurement took approximately 10 seconds at 700 mm of the longitudinal translation along the back. The single point measurement accuracy was 0.1 mm. Computer analysis of the measured surface returned two 3D curves. The first curve was determined by manual marking (manual curve), and the second was determined by detecting surface curvature extremes (automatic curve). The manual and automatic curve comparison was given as the root mean square deviation (RMSD) for each patient. The intra-operator study involved assessing 20 successive measurements of the same person, and the inter-operator study involved assessing measurements from 8 operators.

**Results:**

The results obtained for the 24 patients showed that the typical RMSD between the manual and automatic curve was 5.0 mm in the frontal plane and 1.0 mm in the sagittal plane, which is a good result compared with palpatory accuracy (9.8 mm). The intra-operator repeatability of the presented method in the frontal and sagittal planes was 0.45 mm and 0.06 mm, respectively. The inter-operator repeatability assessment shows that that the presented method is invariant to the operator of the computer program with the presented method.

**Conclusions:**

The main novelty of the presented paper is the development of a new, non-contact method that provides a quick, precise and non-invasive way to determine the spatial spine curve for patients with developed scoliosis and the validation of the presented method using the palpation of the spinous processes, where no harmful ionizing radiation is present.

## Background

Human upright posture analysis is of key importance in medicine because incorrect posture may be a reason for a vast number of pathological conditions [1]. An anatomical expression of the so-called double »S« shaped sagittal curvatures is one of the measures for a correct body posture in clinical assessment [2]. A number of problems can change the structure of the spine or damage the vertebrae and its surrounding tissue, including infections, injuries, tumors, bone changes that develop with age such as spinal stenosis, herniated disks and conditions such as ankylosing spondylitis and scoliosis [3]. Because identified pathological curvatures of the spine are a consequence of the aforementioned problems, clinicians prefer obtaining a quantitative assessment of human posture using one of the numerous measuring methods.

Body posture can be assessed using methods that determine the internal deformity of the torso or assess the external shape of the torso. The internal deformity assessment can be performed using radiographic imaging methods, such as magnetic resonance imaging (MRI) and radiography, or ultrasound-based imaging methods such as medical ultrasonography. However, the main limitations of the MRI imaging methods are their high costs and low availability [4], whereas the radiographic imaging method is known to be harmful to patients due to its cumulative effect of ionizing radiation [5]. The major drawbacks of the medical ultrasonography method are linked to speckle noise [6] and its relative dependence on a skilled operator [7]. The external shape of the torso can be determined using several methods, most commonly mechanical methods such as the DeBrunner kyphometer [8], »Flexicurve ruler« [9], Gravity goniometer or inclinometer [10] and Myrin inclinometer [11]. Optical methods such as raster stereography [12], Moiré topography [13] and laser triangulation imaging [14] are also used. The main drawback of mechanical methods is the lack of automated processes such as data storing, processing and visualization.

To avoid all of the aforementioned disadvantages, numerous efforts have been focused on the development of an alternative optical 3D imaging method that has a low cost and high speed and is accurate [15]. One of the most important objectives of optical metrology in recent years has been to replace evaluations that are based on radiography [16]. The second important objective is the possibility of acquiring, storing, processing and visualizing the measured 3D data [17]. Therefore, over the last 2 decades, many 3D imaging systems and methods for analyzing human torsos with developed scoliosis have been established.

Scoliosis is an abnormal 3D curvature of the vertebral column accompanied by asymmetry and deformities of the external surface of the trunk [18]. It involves elemental deformities in the three main anatomical planes: lateral curvature in the frontal plane, anteroposterior (lordotic and kyphotic) deviation in the sagittal plane, and vertebral axial rotation in the transverse plane [19,14]. In modern clinical practice, X-ray acquisition is recommended every 6 months until the patient reaches the age at which growth stops [20]. The demand for an accurate and reliable clinical evaluation of the spatial spine curve in scoliosis is hardly new because there are many studies of spine curve determination based on the internal torso deformity assessment and the external shape of the torso assessment.

The identification of spatial spine curve classification patterns of the scoliosis spinal deformity was studied by Hong [21]. A 3D spine model was constructed based on the frontal and sagittal X-ray images. The spatial spine curves were extracted from the 3D Bezier curves with 17 uniformed segments and 18 nodes that were superimposed over the X-ray images. A similar study in which the spatial spine curves were obtained from the set of middle control points of each vertebral body on the X-ray images was performed by Devedžić et al. [22]. Ranavolo et al. [23] determined the sagittal spine curve, which was obtained by calculating the coordinates of the vertebral centroids from the lateral radiographs. Centroids were defined as the intersections of the diagonal lines on each vertebra. They discovered that the sagittal spine shape can be determined by 5^th^-order polynomial interpolation.

The trunk surface topography, which was acquired using a laser triangulation imaging system to predict the scoliosis curve type using support vector machines, was analyzed by Assi et al. [24]. The spatial spinal curve extraction was a polygonal line located along the commonly named back valley, which is one of the most visible features of the trunk external surface. Seoud et al. [16] used the same system to determine the spatial spine curve by applying the section extraction approach to the 3D torso shape. The curve on the back that joins the vertebral prominence was represented by cubic splines that were equally spaced along the curve by 100 points. Another interesting approach, in which the spatial spine curves were determined using the volume decomposition routine, was presented by Ajemba et al. [25]. In this routine, a regular set of cross sections of the torso was obtained, and the centroid line of the trunk was computed. The cross section sets and centroid lines were interdependent. The final centroid line was computed interactively, considering the inclination of the torso.

There is occasionally a need to use two independent imaging systems, one to acquire the internal torso deformity and the other to acquire the external shape of the torso. Jaremko et al. [26] used the laser triangulation system for 3D torso surface acquisition and postero-anterior X-ray imaging simultaneously. Data from both systems were combined to yield a superimposed 3D torso spine model. The determination of spatial spine curve was performed by visual estimation based on the selection of the best spinous process locations, which were characterized by bumps and dips on the back surface. The range of spine curve lateral deviation between the spinous process levels T12-L4, based on 48 scans, was 9.1 mm ± 4.9 mm. Validation of the raster stereographic imaging method using the radiographic imaging method was investigated by Hackenberg et al. [12]. Raster stereographic and radiographic frontal spine curves were compared by best-fit superimposition. They found that accuracy of raster stereography, based on 25 patients and measured using root-mean-square differences, was 5.0 mm.

A review of the literature shows that the spatial spine curve can be determined by both internal deformity torso assessment and external torso shape assessment. However, to the best of the authors’ knowledge, there are no reported studies using the laser triangulation imaging method to automatically determine the spatial spine curve and validate the method by spinous process palpation instead of using harmful and invasive radiographic imaging methods. The main novelty of the presented method over introduced [4-14] is the automatic determination of the spatial spine curve of the measured 3D shape of the back. Additionally, a new validation method based on the determination of the manual spine curve, defined by palpation of the thoracic and lumbar spinous processes is presented.

## Methods

### Measuring principle

**Figure 1:**
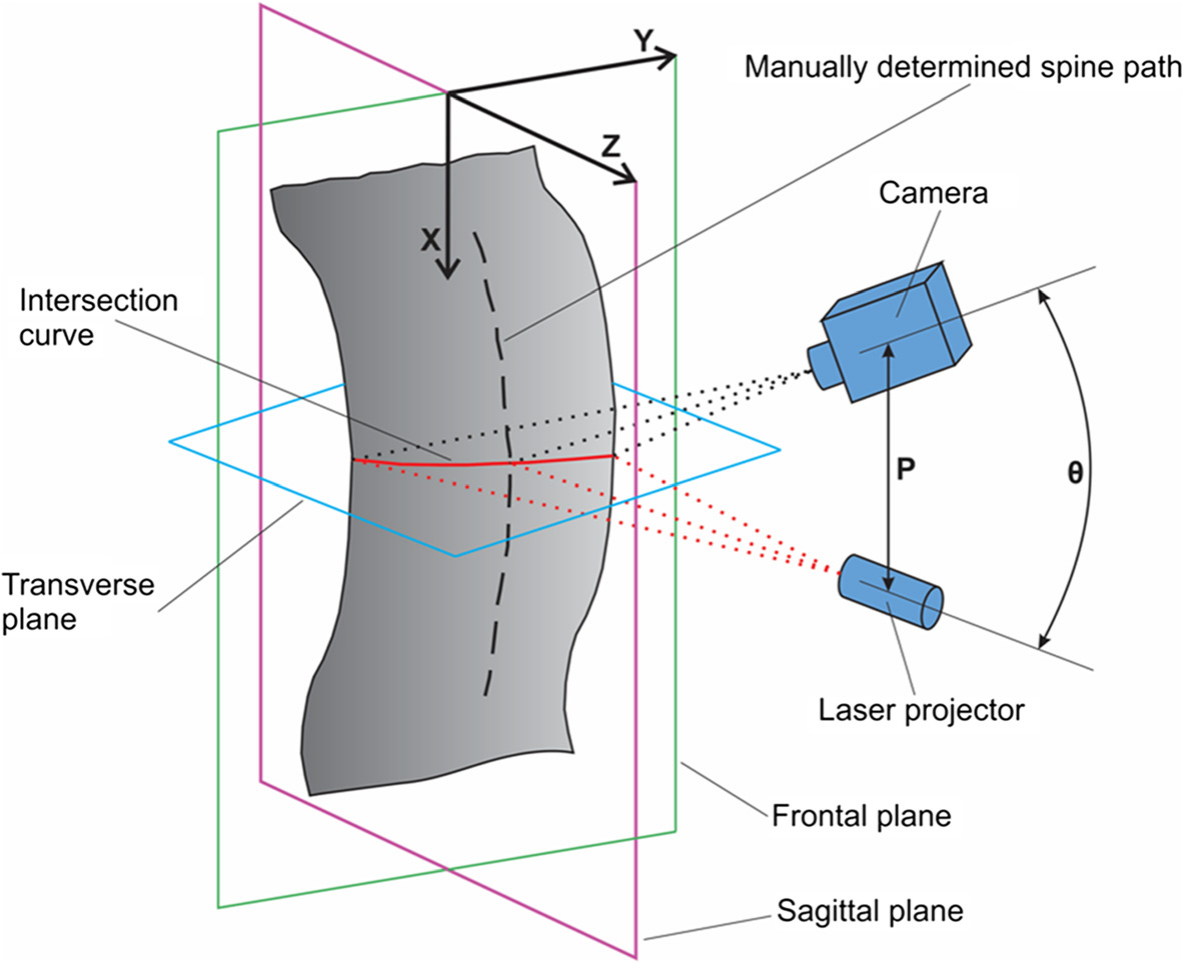
**The triangulation measurement scheme.** Principle of one-laser-plane triangulation and the three main anatomical planes.

Three-dimensional measurement of the human backs was performed using the 3D laser triangulation principle [27]. It is based on the translational movement of a laser across the measuring area, where the measuring area is illuminated by a single laser light plane. Two basic elements of the specially developed laser triangulation system are a grayscale camera and a laser line projector, as shown in Figure 1. The camera (A301f, Basler, Ahrensburg, Germany) contains a 1/2″ CCD image sensor, has a resolution of 658 x 494 pixels and a maximum frequency of video acquisition of 80 Hz, and it was connected to a PC via the FireWire bus. The laser line projector, with 5 mW of power and a wavelength (λ) of 670 nm (red), generated one laser plane that was directed to the measured human back through optical elements. The intersection of the laser plane with the measured surface presents an intersection curve, which was observed under different viewing angles by the camera. Because the distance between the camera and the laser source P and their mutual angle θ was known, the position of the intersection curve in 3D space could be calculated using the triangulation method. Thus, a point cloud was acquired and used for further processing.

To capture the entire shape of the human back, the assembly of the laser projector and the camera had to be moved along the X direction using a computer-controlled linear translator. The measuring speed of intersection curves along the back was approximately 80 profiles per second. The measurement took approximately 10 s for 700 mm of the longitudinal translation. The minimum distance between two adjacent measured profiles (measurement resolution) depended on the translation velocity and measurement frequency and was approximately 0.9 mm. The measuring range was 300 × 700 × 500 mm (width × height × depth) at a distance of 1 m. After the calibration, the accuracy of single point measurement was 0.1 mm [28].

Custom software was developed for the presented laser triangulation system, which allowed us to determine the intersection curves with a sub-pixel resolution in real time during the translational movement of the system along the back. After the measurement was completed, the measured surface, which is presented as an ordered point cloud, was stored. For each point of intersection curve, the corresponding spatial (X, Y, and Z) coordinates and brightness information (in an image coordinate system u, v) was stored.

### Measurement analysis

Twenty-four patients (mean age 40.3 years; range 16–82; 21 females and 3 males) with confirmed clinical diagnosis of scoliosis were scanned using the 3D laser profilometer. The measurement protocol was the same for each patient. The patients were measured in the upright standing posture, leaning against foam attached to the wall with their arms placed by the body. Each patient held their breath during each measurement, which lasted approximately 10 seconds.

Measurements were performed at the University Medical Centre Maribor. The National Medical Ethics Committee of the Republic of Slovenia made a positive declaration and approved the realization of the presented research. All patients consented to participate in the presented research after obtaining an informed written consent. In case of children participation in the research, the informed written consent was obtained either by their parent or guardian.

The research topic was to compare the manual curves, obtained from palpation and automatic spine curves, obtained from the 3D depth image. The algorithms developed allow us to analyze spine curves in all three main anatomical planes, i.e., the frontal (X-Y), sagittal (X-Z) and transverse (Y-Z) plane (Figure 1). Since physicians find the most interesting spine curves in the sagittal and frontal planes, therefore we projected the spatial spine curve (3D) to the frontal (X-Y) and sagittal (X-Z) view. In the following pages, the individual steps of the algorithms for spatial determination of the human spine curve are described.

**Figure 2:**
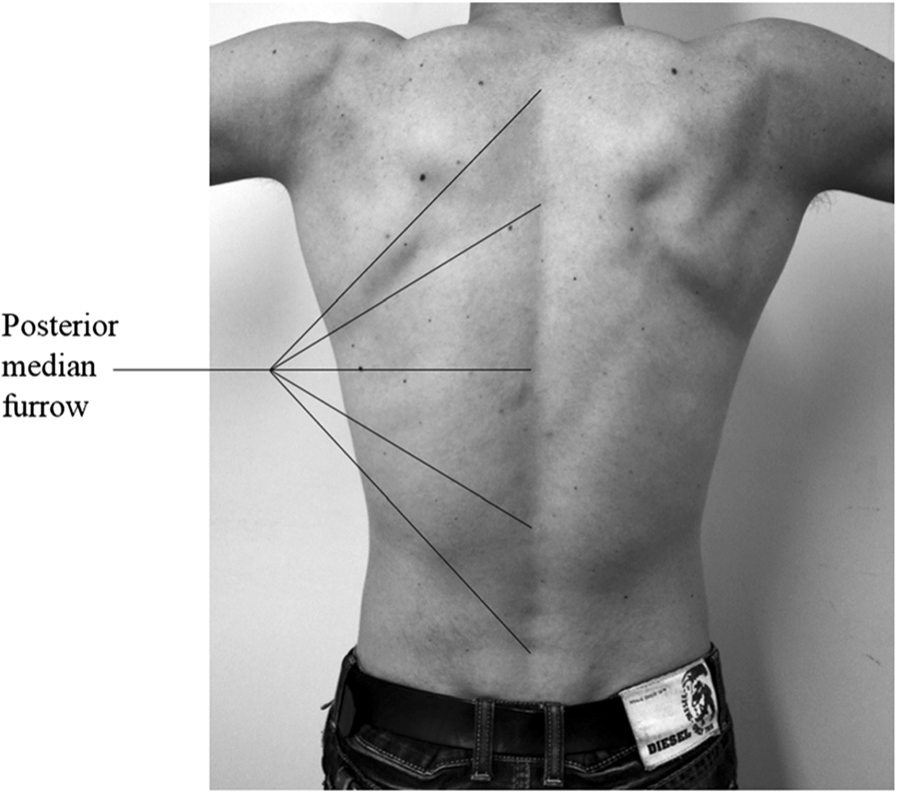
**Morphological features of the human back.** Posterior median furrow on the surface of an average human back.

If we observe a human back in the frontal plane, it can be noted that in the middle of the back there is the so-called posterior median furrow, which is defined as a midline longitudinal depression on the surface of the back [29] as shown in Figure 2. The furrow overlies the tips of the individual vertebrae’s spinous processes, which can be palpated. Therefore, the palpated line of spinous processes at the surface of the back presents a possible method for clinical spine curve determination. In the cervical region, the furrow is superiorly curved, whereas the furrow is deepest in the lower thoracic and upper lumbar parts of the spine. When standing in an upright position, the furrow in the lumbar region is most visible by skin depression because of the vertebral column flexion. Near the sacrum, in the flattened triangular area, the furrow ends and is replaced by the intergluteal cleft. The algorithm for the spatial spine curve determination was therefore based on the known morphological features of the human back presented above.

The main objective of the 3D analysis of the human back was to obtain a spatial curve of the thoracic and lumbar spine based on the measured 3D shape of the back. The spine curve determined through this method was defined as an automatic curve.

**Figure 3:**
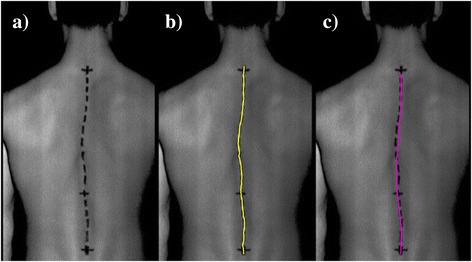
**Marked spinous processes, manual and automatic spine curve. (a)** A grayscale image of the marked spinous processes, as drawn by the physician. Transition spine zones are marked by additional black horizontal lines; **(b)** an example of a manually determined spine curve (named the manual curve); **(c)** an example of an automatically determined spine curve based on the measured 3D shape of the back (named the automatic curve).

Manual determination of the spine curve was performed by palpation of the thoracic and lumbar spinous processes and by marking palpated spinous process with a dark alcohol marker, as shown in Figure 3a. The spine curve obtained using the manual methods was defined as a manual curve. The transitions between cervical and thoracic, thoracic and lumbar, and lumbar and cervical spine were marked with a short horizontal line. The accuracy of thoracic and lumbar spinous processes palpation was assumed to be 9.8 mm [30].

### Manual curve determination

First, the region of interest (ROI) was defined, within which the determination of an automatic and manual curve was performed. Next, the manual curve determination was performed on a grayscale image that represented the brightness of the measured 3D points. The grayscale image was first filtered with a Gauss convolution filter (kernel size: 7 × 7 points) to smooth the wide and high contrast markings. The determination of manual markings was then performed by identifying the minimum pixel intensity along individual image rows and fitting a quadratic polynomial line to sequential groups of data points [31]. With the appropriate settings (intensity threshold detection ranges from 0.08 to 0.15) and one-dimensional interpolation, a continuous 2D curve (placed in an image coordinate system u, v) that presents the manual curve was obtained, as shown in Figure 3b.

### Automatic curve determination

In this step, the 3D depth image of the back was used. The surface curvature along the Y axis was calculated from the depth image using the following well-known expression [32]:

1 $$ K=\frac{\frac{d^2Z}{d{Y}^2}}{{\left[1+{\left(\frac{dZ}{dY}\right)}^2\right]}^{3/2}} $$

**Figure 4:**
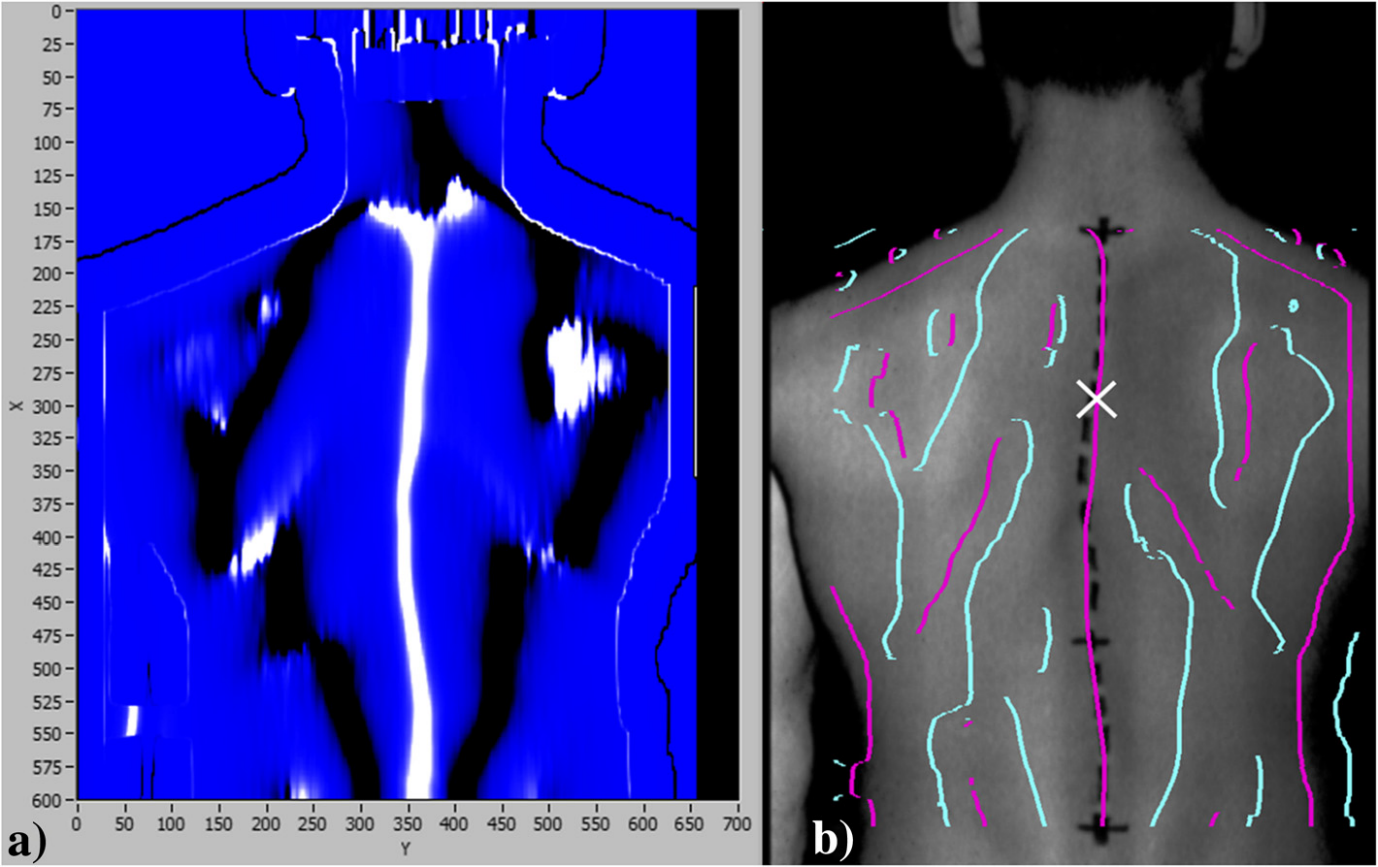
**Back surface curvature calculation and all detected automatic curves. (a)** Calculation of the back surface curvature based on the 3D measurement of the back. The bright areas represent positive curvature, and the dark areas represent negative curvature; **(b)** all detected curves that present possible candidates for the automatic curve. The white cross represents the appropriate curve, which was extracted in a single user mouse click.

The result was invariant to the orientation of the patient around the X axis. An example of the calculation of the back surface curvature is shown in Figure 4a. Positive curvature of the back surface is visible in the white-colored areas, and negative curvature is visible in the dark-colored areas.

**Figure 5:**
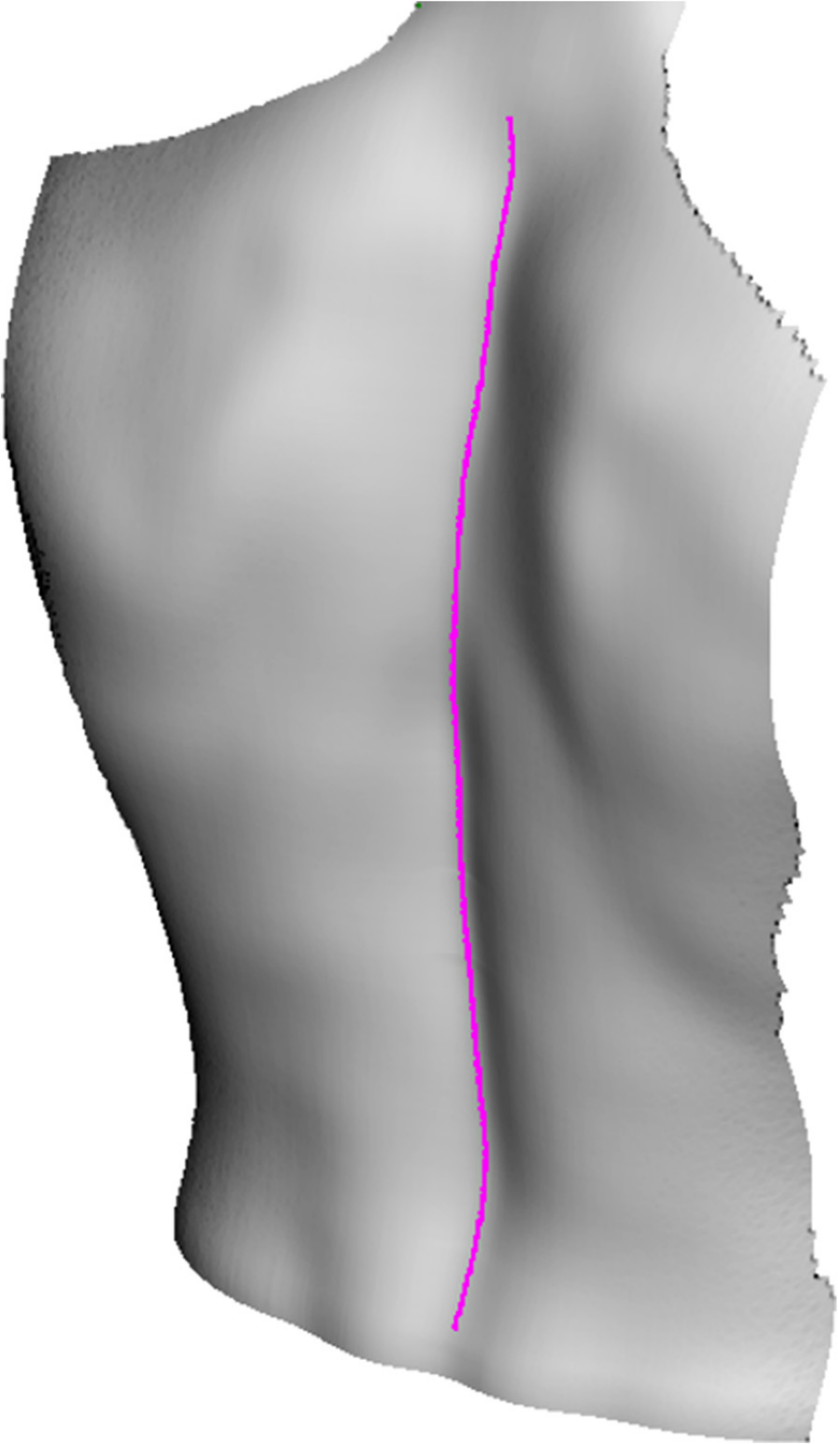
**Example of a determined automatic spatial spine curve.** Determined automatic spatial spine curve overlaying the visualized 3D shape of the back.

Determination of an automatic curve was performed by searching the extremes of curvature along an individual image row (Figure 4a). The technique is based on fitting a quadratic polynomial to sequential groups of data points [31]. The detection threshold for curvature values was set to zero because we want to detect all of the curves that are possible candidates for the automatic curve (Figure 4b). The image coordinates (u, v) of the appropriate curve are then acquired in a single user mouse click (Figure 4b). The procedure occurs next, in which the appropriate spine curve is extracted to determine a continuous 2D curve (placed in an image coordinate system u, v) that represents the automatic curve, as shown in Figure 5.

**Figure 6:**
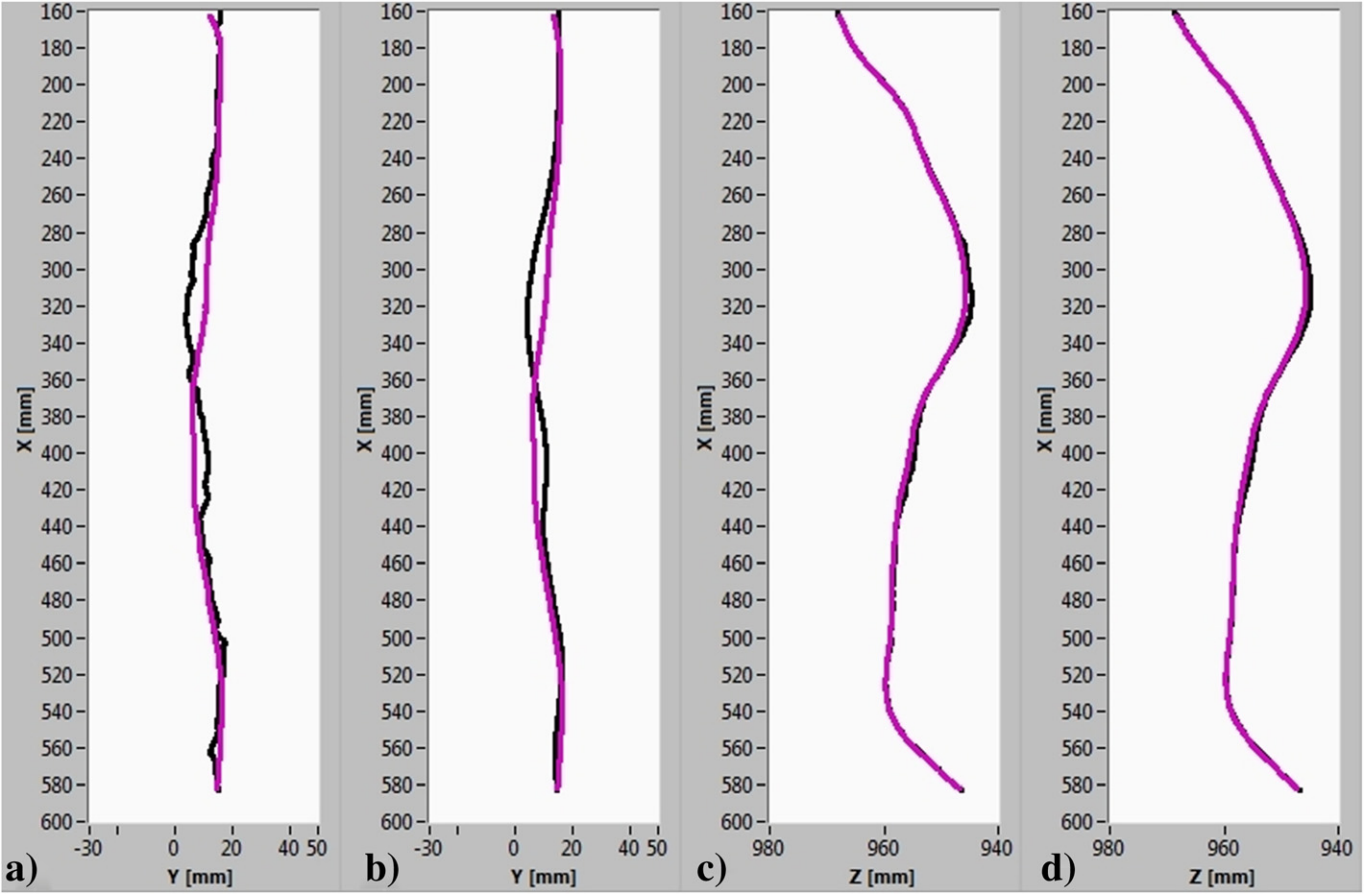
**Spatial manual and automatic spine curve.** Spatial manual curve (black) and spatial automatic curve (purple) in the **(a)** frontal and **(c)** sagittal planes. Cubic spline approximations on both curves in the **(b)** frontal and **(d)** sagittal planes.

To determine the spatial 3D curves, a spatial manual curve and an automatic curve from the 3D depth image of the back were extracted. The extraction was based on the image coordinates (u, v) of the previously detected 2D curves. Thus, both continuous automatic and manual spatial 3D curves that are represented by the X, Y, Z coordinates were obtained. Both curves are shown in the frontal (Figure 6a) and sagittal (Figure 6c) planes.

To smooth the determined spine curves (Figure 6b, d), the cubic spline fitting method was applied [33]. The first reason for choosing the cubic spline approximation over the polynomial approximation was to maintain the morphological features of the back when applying the smoothing to a data set. The curvature of the spine refers to the normal concave and convex curvatures of the entire spine. The typical spine in the sagittal plane has 4 curvatures: cervical, thoracic, lumbar and sacral [34]. In the frontal plane, the normal spine is represented as a straight line [35]. In contrary, different pathologies, such as scoliotic spine curves, have at least 1 curvature in the frontal plane [35]. We must note that our measurements and measurement analyses were focused exclusively on the thoracic and lumbar parts of the spine. The second reason for choosing the cubic spline approximation was to avoid the poor agreement between the measured and approximated curves near the ends of the defined interval. This phenomenon is known as a Runge’s phenomenon, which occurs when interpolating using high-degree polynomials [36].

### Automatic and manual curve comparison

The manual and automatic spatial curves were compared by calculating the root mean square deviation (RMSD):

2 $$ RMS{D}_{X-Y}=\sqrt{\frac{1}{n}{\displaystyle \sum_{i=1}^N}{\left({Y}_{M,i}-{Y}_{A,i}\right)}^2} $$

3 $$ RMS{D}_{X-Z}=\sqrt{\frac{1}{n}{\displaystyle \sum_{i=1}^N}{\left({Z}_{M,i}-{Z}_{A,i}\right)}^2} $$

where M denotes a manual curve and A denotes an automatic curve. The comparison is based on the difference between two single points in the same row of the X axis, representing automatic and manual curve points.

### Inter-operator repeatability

An inter-operator repeatability assessment was conducted by comparing the results from 8 computer software operators (all males, mean age 29.8 ± 8.9 years) who had not used the software with the described algorithms before. All operators analyzed the same measurement. In the process of determining the manual and automatic curve, all of the important parameters in the computer software were fixed for all operators, except for the region of interest and the appropriate spine curve selection in a single user mouse click. Each operator repeated the process of determining an automatic curve three times.

### Intra-operator repeatability

An intra-operator repeatability study was conducted by comparing 20 successive measurements of the same person (male, 34 years old, height 180 cm, weight 87 kg) in the same position. The individual was measured using the same measurement protocol that has been already described. Similar to the inter-operator repeatability assessment, all of the important parameters in the computer software were fixed for all 20 evaluations, except for the region of interest and the appropriate spine curve selection in a single user mouse click. All measurement analyses were performed by the same operator.

In the intra-operator assessment, we analyzed the repeatability of the manual spine curve and the repeatability of measured differences between the automatic and manual curves. Both analyses were performed separately for the frontal and sagittal planes.

## Results and discussion

The results of the manual and automatic spatial spine curve comparison and the anthropometric characteristics for 24 patients are presented in Table 1. The typical values of RMSD between the manual and automatic spine curves in the frontal and sagittal planes were 5.00 mm and 1.00 mm, respectively. Despite 5 times greater typical values of RMSD_X-Y_ compared with the typical values of RMSD_X-Z_, the maximum value of RMSD_X-Y_ for all 24 patients did not exceed the error of palpation in the frontal plane (7.75 mm < 9.8 mm). The accuracy of palpation was estimated by examining the width of the spinous processes. The average width of the lumbar spinous processes from L1sp to L5sp was 9.8 mm [30]. In that study, 200 subjects’ CT scans were assessed, and no average width of the thoracic spinous processes was found. However, according to a comparison of the cervical, thoracic and lumbar spinous processes characteristics outlined by Tortora et al. [34], the typical thoracic spinous process is not narrower than the typical lumbar spinous process. Considering this information, the margin of error when palpating the midpoint of the thoracic and lumbar spinous processes could be as great as 9.8 mm in the frontal (X-Y) plane.

**Table 1:** **RMSD between manual and automatic spine curves for 24 patients and their anthropometric characteristics**

**Patient**	**Sex [M/F]**	**Age [years]**	**Height [cm]**	**Weight [kg]**	**BMI [kg/m** ^**2**^ **]**	**RMSD**_**X-Y**_ **[mm]**	**RMSD**_**X-Z**_ **[mm]**
1	F	42	168	83	29.4	**7.68**	**1.34**
2	F	32	156	59	24.2	**2.38**	**0.60**
3	F	34	154	53	22.3	**4.36**	**0.51**
4	M	45	173	57	19.0	**4.04**	**0.51**
5	F	52	175	70	22.9	**7.48**	**1.54**
6	F	50	170	65	22.5	**4.17**	**1.25**
7	M	18	169	65	22.8	**3.32**	**0.53**
8	F	62	154	64	27.0	**4.30**	**2.31**
9	F	69	159	69	27.3	**3.85**	**0.70**
10	M	58	168	82	29.1	**3.56**	**0.69**
11	F	82	158	72	28.8	**7.43**	**0.91**
12	F	17	173	68	22.7	**3.80**	**0.69**
13	F	16	169	58	20.3	**3.37**	**0.75**
14	F	17	167	69	24.7	**7.75**	**0.51**
15	F	55	155	55	22.9	**3.27**	**0.60**
16	F	38	179	52	16.2	**3.78**	**0.66**
17	F	30	172	89	30.1	**5.94**	**0.63**
18	F	49	169	52	18.2	**4.98**	**1.40**
19	F	25	170	54	18.7	**4.39**	**0.83**
20	F	29	167	60	21.5	**3.76**	**0.72**
21	F	52	162	60	22.9	**3.84**	**0.58**
22	F	29	170	72	24.9	**4.54**	**1.24**
23	F	50	164	65	24.2	**4.42**	**0.62**
24	F	16	173	58	19.4	**4.71**	**1.10**
**Mean ± SD**		**40.3 ± 17.8**	**166.4 ± 6.9**	**64.6 ± 9.8**	**23.4 ± 3.7**	**4.63 ± 1.48**	**0.88 ± 0.43**

**Figure 7:**
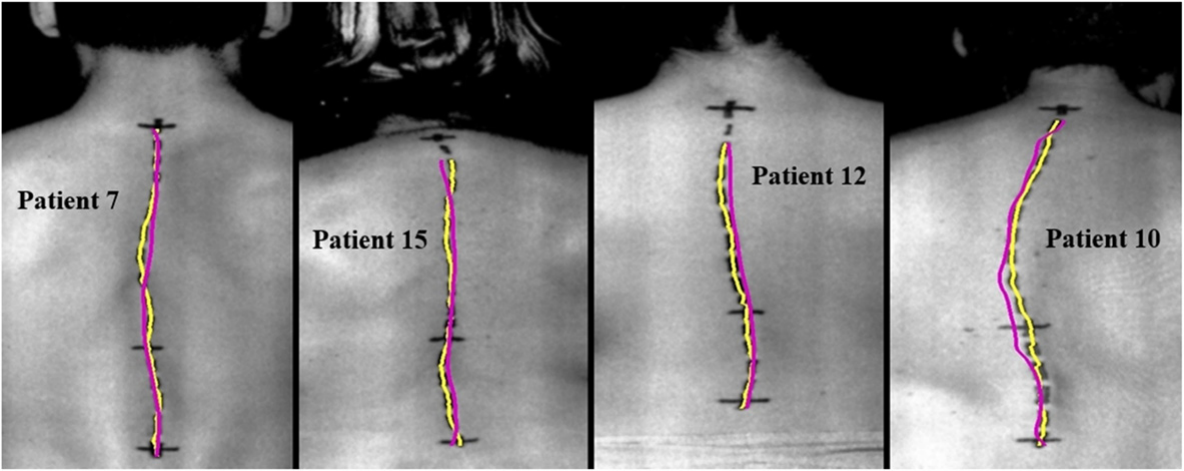
**Example of four determined spatial spine curves.** Determined spatial spine curves, manual (yellow) and automatic (purple), in the frontal plane, as shown for 4 patients.

Four examples of the measured manual and automatic spine curves are shown in Figure 7. Poor overlapping of both curves was likely due to specific characteristics of the patients, such as different skin fold thickness, different physical constitution and different spine curve expression with the positive and/or negative curvature of the surface of the back. The influence of body mass index on the palpating accuracy is reported in [37]. The accuracy of palpation for obese patients at L3sp and L4sp (50% and 44%, respectively) was reported to be significantly lower than the accuracy in non-obese participants (73% and 72%, respectively). Another reason for poor curve overlap could be the transverse movement of the skin in regard to the vertebrae during and after the spinous processes palpation and marking. The problem of skin transverse movement during the patient positioning is reported in [37-39]. In Figure 7, shorter extracted curves in all four cases can also be noted. This result is mainly due to the poor automatic curve detection in the neck region because the depth of the posterior median furrow gradually decreases above the shoulder blades. Hence, the reduction of the region of interest in the X direction must be applied to calculate the RMSD between the manual and automatic spine curves within the same region.

The presented algorithms are included in a semi-automatic program for 3D measurement analysis. The first concern with these algorithms is that to successfully determine automatic and manual curves, the user must define the appropriate region of interest and the appropriate spine curve selection using a single user mouse click. The second concern about the presented algorithms is linked to the patient’s postural sway movements because the average acquisition time of the back is approximately 10 seconds. Thus, the repeatability of measurements for these two aspects needed to be assessed.

**Figure 8:**
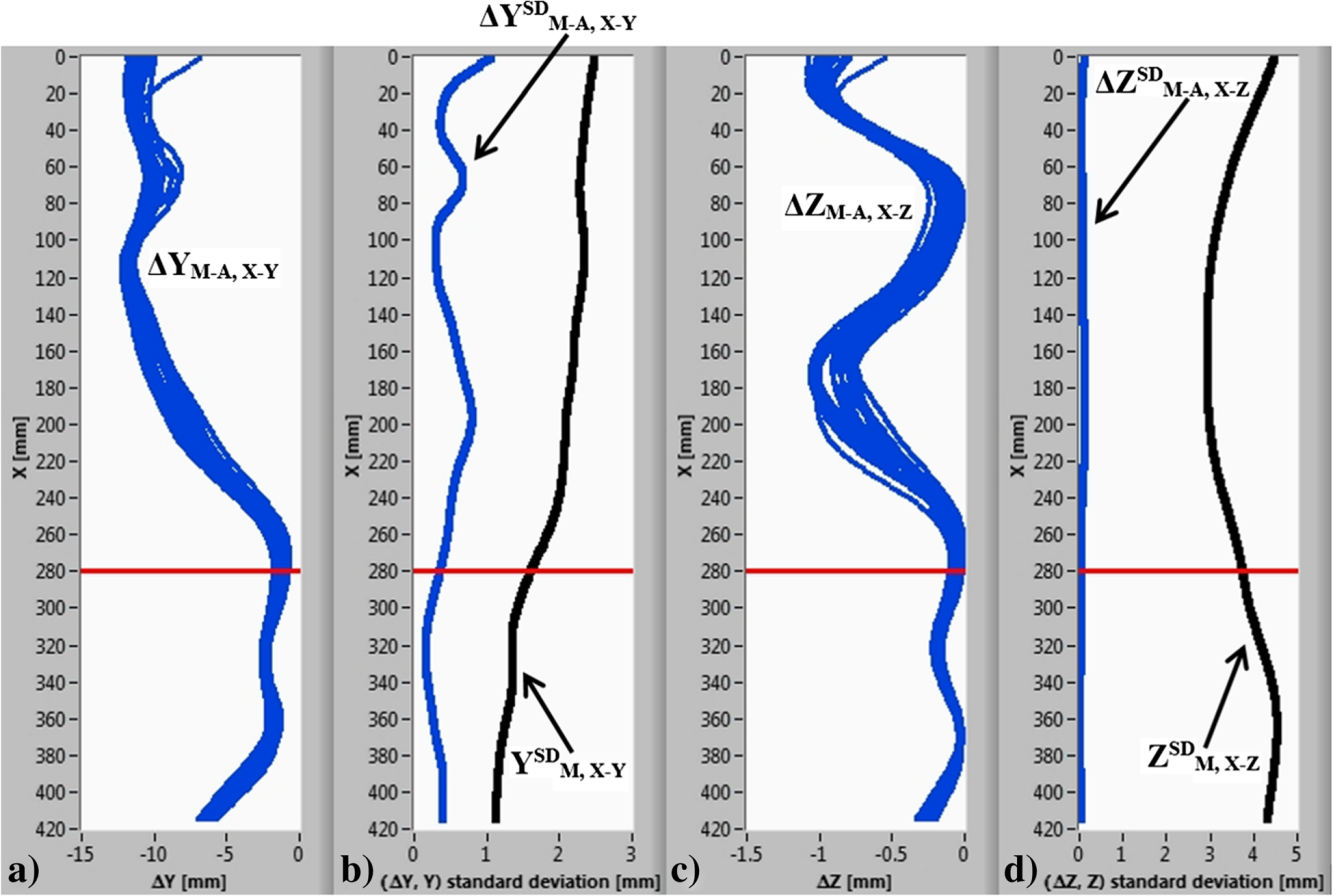
**The intra-operator repeatability results. (a)** A cluster of 20 lines representing the difference between the manual and automatic curves in the frontal plane; **(b)** standard deviation of the differences (blue curve) and standard deviation of 20 manual curve determinations (black curve) at each X coordinate in the frontal plane; **(c)** the differences between the manual and automatic curves in the sagittal plane; **(d)** standard deviation of the differences (blue curve) and standard deviation of 20 manual curve determinations (black curve) at each X coordinate in the sagittal plane. The red horizontal line represents the transition between the thoracic and lumbar spine zones.

The intra-operator repeatability results based on 20 successive measurements are shown in Table 2 and Figure 8. For the frontal plane, a cluster of 20 lines representing the manual and automatic curves differences ΔY_M-A, X-Y_ are given in Figure 8a. In Figure 8b, a standard deviation of ΔY_M-A, X-Y_ at each X coordinate - ΔY^SD^_M-A, X-Y_ is shown with blue curve, which does not exceed 1.00 mm. The black curve Y^SD^_M, X-Y_ in Figure 8b represents the standard deviation of 20 manual curve determinations at each X coordinate, which does not exceed 2.50 mm. Similarly, in the sagittal plane, the differences in the manual and automatic curves ΔZ_M-A, X-Z_ are shown in Figure 8c. The standard deviation of ΔZ_M-A, X-Z_ at each X coordinate - ΔZ^SD^_M-A, X-Z_ in Figure 8d does not exceed 0.20 mm. The black curve in Figure 8d represents the standard deviation of 20 manual spine curve determinations at each X coordinate, which does not exceed 5.0 mm. Based on the results in Table 2, it can be noticed that posture variations Y^SD,AVG^_M, X-Y_ and Z^SD,AVG^_M, X-Z_ between 20 consecutive measurements are generally more than four times greater than the manual and automatic curve difference variations ΔY^SD,AVG^_M-A, X-Y_ and ΔZ^SD,AVG^_M-A, X-Z_ in both the frontal and sagittal planes. Based on these results, we can conclude that in both the frontal and sagittal planes, the repeatability of automatic spine curve determination is at least four times better than the repeatability of human upright posture.

**Table 2:** **Average posture variations and variation of the difference between the manual and automatic curves**

**Frontal plane**		**Sagittal plane**	
Y^SD,AVG^_M, X-Y_ [mm]	ΔY^SD,AVG^_M-A, X-Y_ [mm]	Z^SD,AVG^_M, X-Z_ [mm]	ΔZ^SD,AVG^_M-A, X-Z_ [mm]
1.89	0.45	3.64	0.06

The number of consecutive measurements is 20.

The transition between the thoracic and lumbar spine zones is represented by the red horizontal line in Figure 8. Figure 8b and d also show that the ΔY^SD^_M-A, X-Y_ and ΔZ^SD^_M-A, X-Z_ (blue curves) are higher in the thoracic spine zone. The main reason for the higher deviations, in our opinion, is the effect of body posture on the relief visibility of the back, particularly the position of arms in regard to the torso. In case of arms placed by the body, the relief of the back is less pronounced, and consequently, the determination of the automatic curve is less accurate.

The inter-operator repeatability results, based on the measurement analysis of the same patient made by 8 operators, are shown in Table 3. In both the frontal and sagittal planes, the differences within RMSD_X-Y_ and RMSD_X-Z_ are insignificant. The results clearly indicate that our method is invariant to operator influence, i.e., the human factor.

**Table 3:** **Inter-operator assessment, measured as the RMSD between the manual and automatic spine curves**

**Operator**	**RMSD**_**X-Y**_ **[mm]**	**RMSD**_**X-Z**_ **[mm]**
	4.60	0.75
Operator 1	4.59	0.74
	4.61	0.75
	4.70	0.76
Operator 2	4.69	0.76
	4.65	0.75
	4.63	0.75
Operator 3	4.67	0.76
	4.65	0.75
	4.58	0.75
Operator 4	4.62	0.75
	4.59	0.75
	4.67	0.75
Operator 5	4.69	0.76
	4.65	0.75
	4.61	0.75
Operator 6	4.64	0.76
	4.65	0.76
	4.68	0.75
Operator 7	4.68	0.76
	4.67	0.75
	4.68	0.76
Operator 8	4.69	0.76
	4.66	0.76
**Mean ± SD**	**4.65 ± 0.04**	**0.75 ± 0.01**

We also note that the determined spatial spine curves are not identical to those determined using the X-ray methods. The main reason for the difference is related to vertebral axial rotation in the transverse plane. However, the spine curve determination in clinical practice is important because the proposed method is simple and avoids harmful X-ray radiation. The most valuable advantages of the presented method are (i) the ability to perform frequent measurements of the human back, which is desired because frequent monitoring of the spine curves, curvatures and angles is recommended in modern clinical practice, and (ii) a simple, fast and noninvasive comparison of different therapeutic methods (kinesiotherapy, electrical stimulation, orthotics) on body posture and spine curvatures.

## Conclusions

The main objective of the presented method was to provide a precise and automatic determination of the spatial curve of thoracic and lumbar spine based on the 3D shape measurement of the human torso. Three-dimensional measurements of the backs were performed using a 3D laser profilometer. Each measurement took approximately 10 seconds for 700 mm of longitudinal translation. After calibration, the single point measurement accuracy was 0.1 mm. Computer analysis of the measured surface returned two 3D curves. The first curve, the manual one, was determined by detecting the manual markings, the second, the automatic one, was determined by detecting surface curvature extremes.

The validation included 24 patients with a clinically confirmed scoliosis. The manual curves were treated as a reference curves. They were marked on each patient by palpation of the thoracic and lumbar spinous processes. The results show that the typical RMSD between the manual and automatic curves was 5.0 mm in the frontal (X-Y) and 1.0 mm in the sagittal (X-Z) plane. The results of the inter-operator assessment show that the presented method is invariant to the operator. The intra-operator repeatability of the presented method based on 20 successive measurements of the same subject in the frontal and sagittal planes was found to be 0.45 mm and 0.06 mm, respectively.

The main novelty of the presented method is the developed validation process, which was based on the palpation of the spinous processes. The comparison between the manual curve, determined by palpation and automatic curve, determined from the 3D shape of the back was performed. The proposed method shows great potential and could be used as an alternative to the commonly used X-ray methods, thus allowing for the safer determination of the human posture in a medical setting.

### Consent

Written informed consent was obtained from patients for publication of this paper including accompanying images.

## Abbreviations

MRI: Magnetic resonance imaging
3D: Three-dimensional space
CCD: Charge-coupled device
ROI: Region of interest
RMSD: Root mean square deviation
M-A: Manual-automatic
M: Manual
SD: Standard deviation
AVG: Average
